# Engineering next‐generation crops through CRISPR‐mediated horizontal gene transfer

**DOI:** 10.1111/nph.70951

**Published:** 2026-02-01

**Authors:** Madhab Kumar Sen, Amit Roy, Rajeev K. Varshney, Amrita Chakraborty

**Affiliations:** ^1^ Department of Agroecology and Crop Production, Faculty of Agrobiology, Food and Natural Resources Czech University of Life Sciences Prague Kamýcká 129 16500 Praha‐Suchdol Czech Republic; ^2^ Faculty of Forestry and Wood Sciences Czech University of Life Sciences Prague Kamýcká 129 16500 Praha‐Suchdol Czech Republic; ^3^ State Agricultural Biotechnology Centre, Centre for Crop and Food Innovation, Food Futures Institute Murdoch University Murdoch WA 6150 Australia

**Keywords:** crop engineering, deep learning, horizontal gene transfer, microbial genes, multi‐trait resilience, precision agriculture, synthetic biology

## Abstract

Crops increasingly face overlapping stresses such as heat, drought, salinity, and pathogens that conventional breeding or genome editing rarely overcome in combination. To address this, we propose CRISPR‐enabled horizontal gene transfer (CRISPR‐HGT) as a programmable framework that recreates the evolutionary process by which plants historically acquired adaptive microbial genes. Microbial genes, refined under extreme environments, provide a naturally preadapted resource for multi‐trait resilience. By integrating tools such as Cas12a, CasΦ, RNA‐targeting, and dCas‐based epigenome editors with AI‐guided microbial gene discovery, CRISPR‐HGT enables modular and inducible stress regulation. This approach shifts genome editing from allelic modification to evolution‐guided design. We outline a conceptual pipeline spanning microbial gene mining to adaptive field deployment, highlighting the ecological, biosafety, and regulatory dimensions, from the European Union's cautious oversight to the UK's product‐based framework. CRISPR‐HGT thus introduces an evolution‐informed paradigm for engineering crops that anticipate stress and sustain yield under climate uncertainty.

## Microbial genes as drivers of crop resilience

Crops are now exposed to increasingly complex and concurrent environmental stressors, such as drought, heat, salinity, and pathogen pressure, that rarely occur in isolation. Yet, most breeding and biotechnological interventions continue to address these threats one at a time, an approach that risks obsolescence in the face of accelerating climate volatility (Haber *et al*., 2024). A landmark example of synthetic horizontal gene transfer (HGT) in agriculture is the development of Bt crops, which incorporate a *Bacillus thuringiensis* gene enabling the production of insecticidal proteins effective against major pests, such as the cotton bollworm and European corn borer. Since their commercialisation, Bt crops have significantly reduced reliance on chemical pesticides while increasing yields and lowering environmental impact (Tabashnik & Carrière, 2017). Importantly, they demonstrated the translational potential of microbial genes in crop improvement, establishing a foundation for more sophisticated applications involving gene stacking and multi‐trait resilience.

Building on this precedent, we propose that naturally evolved microbial genes, many mediating functions, such as detoxification, protein stabilisation, and immune modulation, represent a largely untapped genetic resource. With advances in genome editing, synthetic biology, and machine learning–assisted gene discovery, these HGT‐derived elements can now be systematically mined, prioritised, and integrated to engineer crops with durable, multi‐stress resilience.

## Evolutionary aspects of microbial footprints in the plant genome

**Fig. 1:**
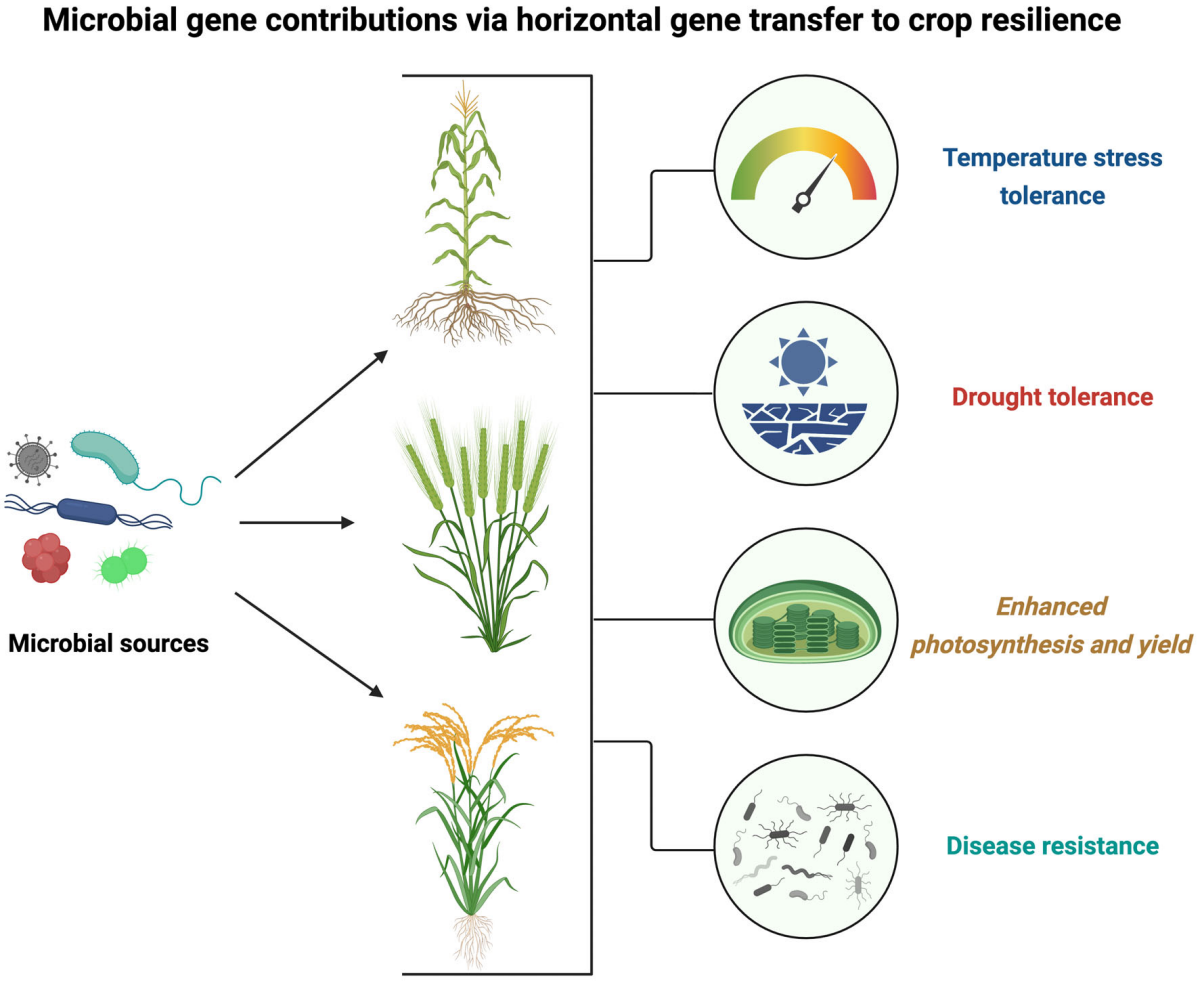
Visual representation of microbial gene transfer and stress resilience traits in crops. This figure was created in BioRender (https://BioRender.com/9are1fl).

Once considered rare in plants, HGT is increasingly recognised as a mechanism by which key adaptive traits have been acquired across evolutionary time (Box 1; Fig. 1). Recent studies reveal that several stress‐resilience traits in land plants may have originated from ancient gene transfers from bacteria or fungi, highlighting the long‐standing biological compatibility between microbial genes and plant systems (Prasad *et al*., 2022; Keeling, 2024). A striking new study has revealed that wheat (*Triticum aestivum*) and its close relatives in the Triticeae tribe harbour bacterial *cold‐shock protein* (*CSP*) genes acquired through HGT, contributing to enhanced drought tolerance, improved photosynthesis, and grain yield (Wang *et al*., 2025). Previously, a study of the liverwort Marchantia (*Marchantia polymorpha*) pangenome identified genes of fungal origin that were acquired before the divergence of land plants, supporting drought adaptation (Beaulieu *et al*., 2025). Similarly, intertidal red algae such as *Pyropia haitanensis* demonstrate a remarkable environmental adaptation capacity through HGT and symbiotic relationships. Its chromosome‐level genome assembly identified 286 HGT‐derived genes, including two genes linked to heat‐stress tolerance (*sirohydrochlorin ferrochelatase* and *peptide‐methionine [R]‐S‐oxide reductase*). In addition, symbiotic actinobacteria, such as *Saccharothrix* spp. modulate stress‐responsive genes, illustrating the synergy between HGT and microbial symbiosis in resilience (Wang *et al*., 2024). Broader surveys indicate that green plants have acquired at least 23 glycosyl hydrolase families from bacteria and fungi, an expansion that supports cell wall diversification and defence evolution (Kfoury *et al*., 2024). Collectively, these findings highlight the evolutionary significance of microbial genes in enhancing plant resilience. Notably, many horizontally acquired genes regulate core homeostasis, detoxification, and signalling rather than isolated stress pathways, supporting their role in broad‐spectrum resilience (Prasad *et al*., 2022). This raises a central question: can these ancient, naturally preadapted genes be systematically identified and reintroduced into elite crops to confer stacked resistance to both abiotic and biotic stressors? If so, HGT may provide both an evolutionary explanation for past plant resilience and a blueprint for engineering resilience in future crops.

Box 1 **Horizontal gene transfer in plants: evolutionary shortcut or synthetic opportunity?**

Horizontal gene transfer (HGT) enables the movement of genetic material across species boundaries, providing a shortcut for acquiring novel traits through nonconventional inheritance. Once considered a microbial phenomenon, HGT is now recognised as an important, though rare, evolutionary force in plants. Evidence from genome analyses reveals that genes of microbial origin have repeatedly contributed to plant adaptation, particularly to stresses, such as drought, salinity, or heat, by expanding metabolic and regulatory diversity.

Many such events blur the line between evolution and engineering. Ancient endosymbioses that produced mitochondria and chloroplasts represent the most dramatic examples of natural gene transfer. More recent integrations, often involving bacterial or fungal partners, suggest a continual exchange between plants and their microbial environment. These transfers demonstrate that microbial genes can operate compatibly within plant systems and remain functional over evolutionary timescales.

This evolutionary precedent provides a compelling rationale for *synthetic HGT*: deliberately reintroducing or designing microbial genes in crops using genome editing and synthetic biology. Rather than imitating transgenesis, synthetic HGT draws on nature's own logic of cross‐kingdom innovation, treating microbes not merely as external partners but as sources of preadapted genetic modules for plant resilience.

## HGT genes: ‘naturally preadapted genes’ as a crop breeding resource

Microbial genes acquired through HGT and refined under extreme environments represent an untapped reservoir for engineering durable, multi‐trait resilience in crops. Once thought rare, HGT is now recognised as a recurrent force in plant evolution, contributing to stress adaptation and metabolic diversification. Recent studies, such as those identifying ancient gene acquisition events in land plants and stress‐adaptive genes in modern crops like wheat and Marchantia, underscore the adaptive significance of microbial gene uptake in shaping plant physiology and stress tolerance (Ma *et al*., 2022; Beaulieu *et al*., 2025; Wang *et al*., 2025). Such findings suggest that many more hidden HGT events may lie within the genomes of crops and their wild relatives. Plant‐associated microbiomes, particularly endophytes and rhizosphere communities, offer co‐evolved reservoirs of such genetic innovation (Ge & Wang, 2025).

With the increasing affordability of genome sequencing, it is now feasible to mine plant genomes, especially those of wild relatives and extremophile species, for signs of functionally relevant HGT. Targeted bioinformatics pipelines can identify genes of putative microbial origin, particularly those encoding stress‐resilience functions, such as antioxidant defence, ion transport, or protein stabilisation (Mishra *et al*., 2024). Machine‐learning models (e.g. DeepGOPlus and HGTector2) integrated with multi‐omics datasets can now prioritise microbial‐like genes that co‐operate within stress‐responsive pathways, improving candidate selection for experimental testing (Oliver, 2000; Zhu *et al*., 2014; Kulmanov & Hoehndorf, 2020). Adding ecological metadata, habitat salinity, aridity, or soil acidity further contextualises HGT persistence and adaptive relevance (Dmitrijeva *et al*., 2024). Once identified, candidate genes can be tested experimentally using model systems and precision phenotyping platforms to confirm cross‐stress tolerance and network integration. Future efforts should expand from single‐gene discovery towards constructing modular stacks of microbial‐derived traits suitable for breeding or synthetic reintroduction (Toju *et al*., 2018).

Traditionally, crop improvement has relied on selection, hybridisation, and, more recently, targeted editing. However, HGT‐derived genes provide a unique evolutionary advantage: they are naturally preadapted to extreme environments and often remain functional across phylogenetic boundaries. Unlike synthetic genes, these microbial elements have already proven compatible with plant regulatory systems, reducing pleiotropic risk and enhancing trait stability (Husnik & McCutcheon, 2018). This direction links evolutionary genomics and breeding innovation, emphasising microbes as long‐term genetic contributors to plant adaptation. Beyond known CSPs, HGT‐derived genes involved in redox sensing, ribosome protection, and signalling may provide additional resilience traits essential for climate‐ready crops.

## Synthetic HGT via CRISPR: a new evolutionary strategy for crop design

**Fig. 2:**
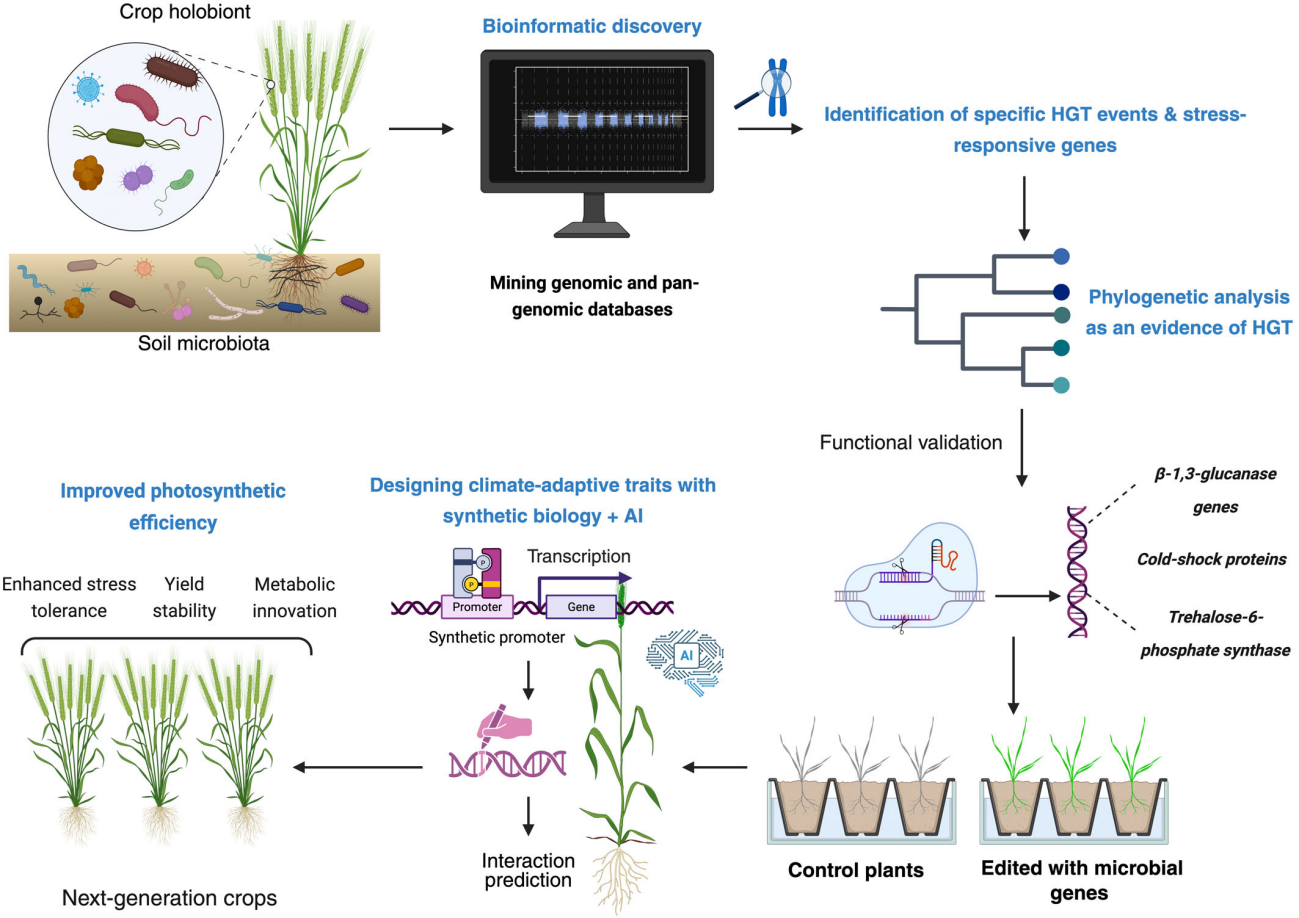
Conceptual model illustrating the integration of bioinformatics, synthetic biology, and artificial intelligence to enhance crop resilience. Microbial genomes serve as reservoirs of stress‐adaptive genes, identified through bioinformatic mining and evolutionary analysis of conserved microbial–plant gene networks. Candidate genes, including those derived from horizontal gene transfer (HGT) events, are functionally validated using CRISPR‐based editing. Synthetic biology and AI tools then optimise these genes for plant systems, enabling precise design of climate‐adaptive traits. The process culminates in the development of resilient crops shaped by microbial diversity and refined through computational intelligence. This figure was created in BioRender (https://BioRender.com/lotx54b).

Building on these evolutionary insights, we propose CRISPR‐HGT, a framework that transforms HGT from a naturally occurring phenomenon into a programmable strategy for crop engineering (Fig. 2). Rather than refining existing alleles, it synthetically reconstructs the process through which plants historically acquired adaptive microbial genes. Using CRISPR systems as molecular mediators, this approach transfers preadapted microbial modules that encode biochemical and regulatory capacities absent from plant genomes. Unlike conventional transgenic pipelines that test bacterial genes individually through empirical optimisation, CRISPR‐HGT integrates artificial intelligence (AI)‐guided gene discovery, modular operon design, and multiplex CRISPR delivery to evaluate microbial modules in parallel. This predictive, system‐level framework reduces empirical trial costs by preselecting gene clusters with validated co‐expression and adaptive relevance, making large‐scale testing more feasible. Two complementary directions can be envisioned: (1) identifying ancient HGT‐derived genes already present in wild relatives and reintroducing them into elite cultivars; and (2) testing synthetic HGT, in which microbial genes currently absent in plants are experimentally integrated and tuned for adaptive performance. This distinction underscores the continuity between natural evolution and its deliberate, CRISPR‐enabled reconstruction. Nevertheless, the first approach using existing HGTs in plants for CRISPR‐HGT can serve as a good starting point for a ‘proof of concept’ study.

To understand how CRISPR‐HGT compares with other crop biotechnology platforms, we outline key distinctions in Table 1. This comparison emphasises how CRISPR‐HGT introduces a multi‐layered, programmable control framework that extends beyond the static or environmentally constrained logic of conventional strategies. However, the central question is whether microbial genes offer real advantages over the immense diversity already existing in plants. We argue that microbial genes complement, rather than replace, plant adaptive potential by providing additional layers of flexibility and expanding the repertoire of resources available for crop engineering. Notably, microbes evolving in highly dynamic and extreme environments, such as those exposed to radiation, desiccation, oxidative stress, and salinity, have been selected for multitrait resilience, which is embedded in compact operons or enzyme clusters (Nwachukwu & Babalola, 2022; Jagadesh *et al*., 2024). These modules integrate detoxification, protein stabilisation, and redox control within autonomous regulatory frameworks, offering a level of biochemical versatility rarely found in plants. Unlike endogenous plant pathways, which are developmentally constrained and stress‐specific, microbial modules are self‐contained and transferable with minimal regulatory conflict. They thus expand the functional space available for crop design, particularly for traits involving novel detoxification or redox buffering chemistry (Raza *et al*., 2023).

**Table 1:** State‐of‐the‐art comparison: positioning CRISPR‐HGT in crop engineering.

Approach	Core mechanism	Functional scope	Control level	Biological limitation
Traditional transgenics (Araus *et al*., 2019)	Stable genomic insertion of isolated genes, often under constitutive promoters.	Single, fixed traits (e.g. pest resistance and herbicide tolerance).	Low. Constitutive expression offers no control over timing, tissue, or environmental responsiveness.	Static function; cannot respond to environmental variability.
Genome editing (Chen *et al*., 2019)	Targeted alteration or CRISPR‐mediated regulation (CRISPRa/i, base editing) of endogenous coding or regulatory sequences.	Trait optimisation within existing gene networks.	Medium–High. Spatial, temporal, or inducible control can be achieved via synthetic promoters or CRISPR regulators.	Constrained by native gene space and existing regulatory architecture.
Microbiome engineering (Arif *et al*., 2020)	Indirect modulation via beneficial microbes affecting host physiology or signalling.	Multi‐trait modulation via microbial consortia.	Low. Control depends on microbial survival, host recruitment, and ecological conditions.	Low predictability, unstable colonisation, and complicated inheritance.
CRISPR‐HGT (proposed)	Precision insertion of microbial genes plus programmable CRISPR control (e.g. CRISPRa/i and epigenome editing).	Modular, multi‐trait stress resilience using novel gene types.	High. Gene activation can be condition‐dependent, tissue‐specific, reversible, and tunable across modules.	Functional integration and regulation are still under development.

While traditional approaches rely on static or environmentally constrained strategies, CRISPR‐HGT offers a programmable, multi‐layered framework for dynamic trait control under complex stress environments. CRISPR‐based tools are increasingly applied across both plant and microbial gene contexts; the distinction here lies in the modular and cross‐kingdom scope of the introduced traits.

Furthermore, recent advances at the interface of CRISPR genome engineering, synthetic biology, and evolutionary genomics now allow evolutionary gene acquisition and functional innovation to be recreated in a programmable manner. Cas12a enables the multiplexed insertion of operon‐like clusters, mirroring natural HGT architectures (Tuncel *et al*., 2025). By contrast, compact nucleases such as Casφ support the delivery of multi‐gene payloads even in large genomes (Li *et al*., 2023). RNA‐targeting systems (Cas13 and CasRx) allow transient modulation under combined stresses (Yang & Patel, 2024), and dCas9/dCas12‐based epigenome editors facilitate reversible gene activation or silencing (Villiger *et al*., 2024; Sen *et al*., 2025). Together, these platforms enable conditional, multi‐layered regulation, allowing microbial genes (i.e. those originating from bacteria, fungi, and archaea) to remain silent under normal growth conditions and activate dynamically in response to stress combinations.

Importantly, CRISPR‐HGT is not proposed to bypass transgenic regulation but to extend the spectrum of genetic sources across biological kingdoms, drawing its conceptual foundation from evolutionary events. While such constructs would remain transgenic, reintroducing genes from microbial origin already present in plant‐associated microbiomes or ancestral lineages could streamline risk evaluation relative to wholly synthetic constructs. The novelty lies in its evolutionary rationale: using microbial ingenuity as a modular toolkit to accelerate the rational design of climate‐resilient crops.

## Ecological trade‐offs in CRISPR‐HGT‐enabled crops

CRISPR‐HGT presents a compelling approach for enhancing crop resilience by incorporating microbial genes. Yet, the durability of such traits will ultimately hinge on eco‐evolutionary trade‐offs that mediate their costs and benefits under shifting environmental conditions. Insights derived from natural instances of HGT, as well as from microbial inoculation studies, underscore this intrinsic duality. While microbial genes can boost resilience under extreme conditions, they may impose energetic or regulatory burdens under optimal growth, echoing the growth–defence trade‐offs familiar in plant–microbe systems (Etesami, 2025). For instance, certain plant growth‐promoting bacteria (PGPB) produce auxins, ethylene, or volatile compounds that enhance plant growth at moderate levels but trigger necrosis or metabolic stress when overproduced (Etesami & Glick, 2024; Etesami, 2025). Analogously, CRISPR‐HGT modules encoding microbial stress metabolites may function beneficially only within narrow environmental windows, with fitness costs emerging under benign conditions.

At a broader eco‐evolutionary scale, HGT‐derived traits could reconfigure plant–biotic and plant–environment interactions. Just as PGPB inoculations sometimes destabilise native microbial communities, HGT traits could shift plant resource allocation or disrupt established symbioses. Introduced bacterial strains have been shown to outcompete arbuscular mycorrhizal fungi, reducing nutrient cycling and ecosystem stability in semiarid regions (Etesami, 2025). Similarly, CRISPR‐HGT modules that alter secondary metabolism or antimicrobial production might strengthen pathogen resistance but inadvertently impair beneficial partnerships. For instance, antibiotics synthesised by PGPB to suppress pathogens can also damage plant cells when drought or salinity limits detoxification capacity (Etesami, 2025). These examples illustrate how context‐dependent trade‐offs may constrain the ecological stability and persistence of synthetic HGT traits across environments.

For agricultural deployment, managing eco‐evolutionary trade‐offs will be critical. Stacking multiple microbial modules could not only strengthen multi‐trait resilience but also increase pleiotropy and metabolic burden. Conditional or stress‐inducible expression systems can mitigate these costs by activating microbial pathways only in response to relevant cues. Insights from natural HGT and microbial inoculation studies indicate that traits conferring advantages only under rare stresses should be conditionally expressed, whereas broadly beneficial traits can support long‐term stability. The success of CRISPR‐HGT will thus depend not only on technical and regulatory progress but also on how effectively these traits integrate into eco‐evolutionary feedbacks. Integrating empirical evidence with predictive modelling using deep learning could guide the design of CRISPR‐HGT systems that are both technologically innovative and ecologically sustainable, as well as evolutionarily supported.

## Scientific and regulatory challenges of CRISPR‐HGT

Despite its transformative potential, the CRISPR‐HGT approach will also face significant scientific and regulatory hurdles. Regulatory support is essential for marketing the CRISPR‐HGT‐derived resilient next‐generation crops. Delivering microbial genes into crops often places such innovations under transgenic classifications in many jurisdictions, triggering stringent approval processes and public scrutiny. In regions such as the European Union (EU) and much of Africa, this would likely subject CRISPR‐HGT products to the most restrictive and costly transgenic regulatory pathways, effectively limiting cultivation or field testing. Concerns about ecological risk, gene flow, off‐target effects, and the stability of complex trait expression across environments remain legitimate and unresolved at scale (Gilbertson *et al*., 2025). Unlike edits to native genes, HGT‐derived modules may encode nonplant proteins or foreign regulatory motifs, introducing risks, such as immunogenicity, network disruption, or unintended metabolic burdens. These risks are less predictable and may differ across environments. It is essential to develop an internationally cohesive regulatory framework in collaboration with scientists and policymakers based on rigorous biosafety and efficacy assessment for CRISPR‐HGT‐derived resilient crops. However, the EU's ongoing regulatory reform process currently represents a major obstacle to such harmonisation, as gene‐edited and transgenic crops continue to face near‐identical oversight. CRISPR‐HGT is not proposed to circumvent transgenic regulation but to align with evolving, evidence‐based frameworks. Because CRISPR‐HGT introduces microbial genes outside natural or sexually compatible variation, such constructs remain under full GMO‐style oversight, with risk assessment guided by whether a trait could arise through traditional breeding (‘Precision bred plants’, 2025). In the near term, evolutionary HGT events already present in plant genomes offer a more feasible and publicly acceptable route, as they can be leveraged through breeding or cisgenic approaches, which are exempt from the most restrictive transgenic oversight. Nevertheless, synthetic HGT constructs, meanwhile, will require rigorous biosafety validation and transparent regulatory engagement before field deployment.

By contrast, the UK's post‐Brexit regulatory vision, outlined by the Regulatory Horizons Council, offers a model for adaptive, innovation‐supportive governance (‘Regulatory Horizons Council report on genetic technologies’, 2022). The Council advocates a shift from method‐ to product‐based regulation, assessing the properties and risks of the final product rather than the genetic technique used to create it. This framework emphasises proportional data requirements, stakeholder engagement, and flexibility through evolving standards rather than rigid legislation, supported by a dedicated risk‐assessment body (ACRE‐2) modelled after the MHRA. Such an approach aims to ensure safety without constraining innovation, yet broad acceptance of CRISPR‐HGT remains premature. Microbial‐gene constructs will continue to undergo thorough ecological scrutiny, particularly regarding biodiversity and gene flow risks. Harmonisation between jurisdictions will depend on accumulating evidence on biosafety. Transparent, publicly funded proof‐of‐concept research with early involvement from the regulator offers the most credible pathway. As the UK and other nations adopt regulatory ‘sandboxes’ for emerging biotechnologies, data‐driven frameworks balancing precaution with innovation may ultimately shape CRISPR‐HGT into a scientifically robust and socially legitimate route for next‐generation crop design.

As developing nations face concurrent yield losses and climate uncertainty, CRISPR‐HGT provides a modular framework for crop innovation that is adaptable across diverse environments. Modularisation of HGT components, integrated with high‐resolution phenotyping and AI‐guided prediction, can optimise trait architecture and minimise risk. Beyond standard breeding support, AI plays a central role in CRISPR‐HGT by predicting the compatibility of microbial modules with plant regulatory and metabolic networks. Emerging tools such as DeepCRISPR (Chuai *et al*., 2018), CRISPRon (Xiang *et al*., 2021), and CRISPR‐PLANT v2 (Minkenberg *et al*., 2019) already enhance guide RNA design and editing precision, while machine learning models trained on multi‐omics and phenotypic datasets can identify microbial genes most likely to integrate functionally, anticipate trade‐offs among stacked traits, and optimise inducible promoter design (Li *et al*., 2025). Coupled with precision phenotyping, AI thus transforms CRISPR‐HGT from a static editing workflow into a predictive system for engineering stable, cross‐kingdom gene–network dynamics. Nevertheless, experimental confirmation of model‐derived insights remains essential before any progression towards field applications. Achieving socially acceptable and ecologically sound CRISPR‐HGT crops will, however, require transparent biosafety innovation and early dialogue among scientists, regulators, and stakeholders. Unlike precision‐bred edits that fall within natural allelic diversity and anticipate conventional‐breeding‐like risk profiles (refer new regulation issued by the Department for Environment, Food & Rural Affairs, UK on 13 November 2025), CRISPR‐HGT constructs introduce inherently novel modules, an important determinant of future ecological assessment, especially in biodiversity‐sensitive domains, such as forestry. In perennial forestry species, these assessments become even more complex. Long generation times, extensive pollen‐mediated gene flow, and deep integration into natural ecosystems mean that microbial‐gene constructs must be evaluated over longer ecological timescales than annual crops. Moreover, containment and postrelease monitoring are significantly more challenging in trees, where traits may spread across landscapes before ecological impacts can be fully realised. These forestry‐specific considerations will likely require modified risk‐assessment frameworks and extended field evaluations.

## Concluding remarks and future outlook

CRISPR‐HGT aids crop improvement by transforming microbial evolution into a programmable design tool for resilience. By treating microbial genes as functional partners rather than foreign insertions, this framework expands the genetic repertoire available for building resilience to complex, overlapping stresses. Integrating precision genome editing, synthetic biology, and AI‐guided discovery will accelerate the identification and optimisation of microbial modules suited for future climates. Equally, advances in biosafety, transparent regulation, and context‐specific expression control will ensure that innovation proceeds responsibly. The next frontier lies in translating CRISPR‐HGT from concept to field, testing its capacity to create crops that anticipate stress, adapt dynamically, and sustain yield under environmental uncertainty. This transition marks a shift from incremental improvement to evolution‐informed design, positioning CRISPR‐HGT as a blueprint for building next‐generation resilient crops.

## Competing interests

None declared.

## Author contributions

MKS conceived and designed the research, wrote the initial draft, created the figures, and also prepared the revised manuscript. AR, RKV and AC contributed to discussions, provided critical feedback, and approved the final version.

## Disclaimer

The New Phytologist Foundation remains neutral with regard to jurisdictional claims in maps and in any institutional affiliations.
